# Evidence for the existence of facilitatory interactions between the dopamine D2 receptor and the oxytocin receptor in the amygdala of the rat. Relevance for anxiolytic actions

**DOI:** 10.3389/fphar.2023.1251922

**Published:** 2023-10-12

**Authors:** Juan Carlos Hernández-Mondragón, Dexter A. Hernández-Hernández, Minerva Crespo-Ramírez, Oscar Prospero-García, Luisa Rocha-Arrieta, Kjell Fuxe, Dasiel O. Borroto-Escuela, Miguel Perez de la Mora

**Affiliations:** ^1^ Instituto de Fisiología Celular, Universidad Nacional Autónoma de México, Mexico City, Mexico; ^2^ Laboratorio de Cannabinoides, Departamento de Fisiología, Facultad de Medicina, Universidad Nacional Autónoma de México (UNAM), Mexico City, Mexico; ^3^ Department of Pharmacobiology, Centro de Investigación y Estudios Avanzados (CINVESTAV, Sede Sur), Mexico City, Mexico; ^4^ Department of Neuroscience, Karolinska Institutet, Stockholm, Sweden; ^5^ Receptomics and Brain Disorders Lab, Department of Human Physiology, Faculty of Medicine, University of Malaga, Málaga, Spain

**Keywords:** G protein-coupled receptors, oligomerization, heteroreceptor complexes, oxytocin receptor, dopamine D2 receptor, amygdala, anxiety

## Abstract

**Introduction:** The amygdala is a limbic region of high value for understanding anxiety and its treatment. Dopamine D2 receptors (D2Rs) and oxytocin receptors (OXTRs) have both been shown to participate in modulating anxiety involving effects in the amygdala. The goal is to understand if D2R–OXTR heterocomplexes exist in the central amygdala and if, through enhancing allosteric receptor–receptor interactions, may enhance anxiolytic actions.

**Methods:** The methods used involve the shock-probe burying test, the *in situ* proximity ligation assay (PLA), image acquisition and analysis, and the BRET2 assay. Bilateral cannulas were introduced into the amygdala, and the effects of the coadministration of oxytocin and the D2R-like agonist quinpirole into the amygdala were studied.

**Results:** The combination treatment enhanced the anxiolytic effects compared to the single treatment. The D2R/D3R antagonist raclopride blocked the effects of the combination treatment of oxytocin and the D2R agonist, although oxytocin is regarded as a distinct modulator of fear-mediating anxiolytic effects. *In situ* PLA results indicate the existence of D2R–OXTR heteroreceptor complexes and/or the co-location of OXTR and D2R within the same cell membrane nanodomains in the central amygdala. With BRET2, evidence is given for the existence of D2R–OXTR heteromers in HEK293 cells upon co-transfection.

**Discussion:** The enhanced behavioral effects observed upon co-treatment with OXTR and D2R agonists may reflect the existence of improved positive receptor–receptor interactions in the putative D2R–OXTR heterocomplexes in certain neuronal populations of the basolateral and central amygdala. The D2R–OXTR heterocomplex, especially upon agonist co-activation in the central amygdala, may open a new pharmacological venue for the treatment of anxiety.

## 1 Introduction

The amygdala is central to anxiety regulation and can become hyperactive in response to stress. This heightened activity is linked to the dopaminergic system, causing increased dopamine release. Catecholamine-mediated neurotransmission through the activity of amygdaloid dopamine D2 receptors (D2Rs) has been implicated in modulating fear and anxiety (Pezze and Feldon, 2004; de la Mora et al., 2010; Zarrindast and Khakpai, 2015). Thus, it has been demonstrated that local infusion of D2R antagonists triggers both anxiogenic nonconditioned responses (Perez de la Mora et al., 2012) and a reduction in contextual conditioned fear (de Souza Caetano et al., 2013). PET imaging shows that elevated D2R levels contribute to amygdala dysfunction under stress (Yan et al., 2021). Dopamine also influences memory, helping distinguish safety from threat. Modulating D2Rs in the central amygdala plays a vital role in fear response consolidation, with stimulation aiding discriminative learning and blockade of inducing generalized threat responses (De Bundel et al., 2016).

Oxytocin (OXT) is a well-known nona-neuropeptide that is released from nerve terminals in the posterior pituitary into the bloodstream. Its primary functions include regulating uterine contractions during parturition, facilitating social bonding, and promoting milk let-down during nursing (Jurek and Neumann, 2018). The synthesis of OXT neuropeptide, released in the posterior pituitary, occurs predominantly in the magno- and parvocellular paraventricular hypothalamic neurons as well as in the supraoptic neurons (Onaka and Takayanagi, 2019). It should be noted that species variations exist; for instance, in voles, OXT is also expressed in the bed nucleus of the stria terminalis (Ross and Young, 2009) and in glutamatergic neurons of the human prefrontal cortex (Zhong et al., 2022). The oxytocin neuropeptide binds to its receptor, the oxytocin receptor (OXTR), a G-protein-coupled receptor (GPCR), expressed in the periphery and central nervous system (Jurek and Neumann, 2018; Froemke and Young, 2021). Oxytocin neurons from the hypothalamus project axon collaterals and send projections to various brain regions that possess OXTRs. This suggests that oxytocin’s influence extends to complex behaviors, including those related to food intake, social interactions, and emotional responses (Jurek and Neumann, 2018; Froemke and Young, 2021). The limbic system, central and basolateral amygdala, midbrain serotonin neurons, and other regions of the lower brainstem, including projections into the spinal cord, are among the areas involved in oxytocin signaling (Eliava et al., 2016). Additionally, oxytocin neurons have been found in extrahypothalamic regions (Knobloch and Grinevich, 2014). Overall, oxytocin neurotransmission operates through volume transmission, similar to other neuropeptides, while secreted oxytocin in the bloodstream functions as a peptide hormone (Borroto-Escuela et al., 2015a).

One crucial molecular mechanism that occurs in the cellular plasma membrane is the formation of GPCR heteroreceptor complexes, which can involve dimers or higher-order complexes. These complexes exert allosteric receptor–receptor interactions that modulate the recognition, signaling, and trafficking of the participating receptor protomers, thereby influencing other associated proteins (Fuxe et al., 2010; Borroto-Escuela et al., 2014; Fuxe et al., 2014; Borroto-Escuela et al., 2015b; Fuxe and Borroto-Escuela, 2016; Borroto-Escuela et al., 2017a).

In this work, we aim to understand if dopamine D2 receptor (D2R)–oxytocin receptor (OXTR) heteroreceptor complexes or their co-location within the same cell membrane nanodomains (hereafter described as heterocomplexes) exist in the central amygdala and, through enhancing allosteric receptor–receptor interactions, may produce anxiolytic actions. The methods used involve the shock-probe burying test, the *in situ* proximity ligation assay (PLA), image acquisition and analysis, and the BRET2 assay. Bilateral cannulas were introduced into the amygdala, and the effects of the co-administration of oxytocin and the D2R agonist quinpirole into the amygdala were studied. We will discuss the potential relevance of these complexes in brain function and behavior. The exploration of these heteroreceptor complexes will significantly contribute to oxytocin research, as the allosteric receptor–receptor interactions within OXTR heteroreceptor complexes allow for bidirectional modulation between the OXTR protomer and other participating receptor protomers such as D2R.

## 2 Materials and methods

### 2.1 Drugs

We purchased raclopride, quinpirole, and oxytocin from Sigma Chemical Co. All other drugs and chemicals were obtained from reliable local sources.

### 2.2 Animal experiments

Male Wistar rats weighing between 250 and 270 g, bred at the Instituto of Fisiología Celular, Universidad Nacional Autónoma de México, were used for all the experiments in this study. The rats were housed in a controlled environment with a temperature of 22°C and a light–dark cycle of 12 h (lights on from 6:00 to 18:00). They had unrestricted access to food and water. All experimental procedures were conducted following the ethical guidelines established by the local Mexican Ethics Committee MPM07-14, which adheres to the recommendations provided by the United States Institutes of Health for the Care and Use of Laboratory Animals (NIH Publications No. 8023, revised 1996). The rats were randomly assigned to different groups and tested in a randomized manner. All experiments were performed between 10:00 and 16:00. We made every effort to minimize the number of animals used and reduce their suffering.

### 2.3 Surgery and microinjection

To implant permanent guide cannulas into the central amygdala, the rats were anesthetized with ketamine hydrochloride (100 mg/kg, i. p.) and positioned in a stereotaxic frame (Kopf Instruments, Tujunga, CA, United States) with the incisive bar set at −3.3 mm. It is worth noting that ketamine, used as an anesthetic agent in this study, was unlikely to produce differential effects since it was administered to both the experimental and control rats. During the surgery, the rats’ body temperature was maintained at 37°C using a CMA/150 temperature controller (CMA/Microdialysis, Stockholm, Sweden). Bilateral stainless steel cannulas with an outer diameter of 0.46 mm (C315G, Plastics One, Roanoke, VA, United States) were targeted to the central nucleus of the amygdala (CeA) (coordinates AP: –2.12 mm; L: ±4.7 mm from bregma; V: –7.7 mm from the skull surface) based on the atlas of Paxinos and Watson (1986). The guide cannulas were secured to the skull using stainless steel screws and dental acrylic cement. To maintain patency, dummy cannulas (C315DC, Plastics One) were used. Prior to the surgery, the rats were injected with estreptobezetazil V (1000 IU i. m.) and meloxicam 0.5% (1 mg/kg i. m.) to prevent infections and minimize postoperative discomfort. The animals were individually housed until behavioral testing. For 5 consecutive days, the rats were handled for 5 min per day. After a recovery period of 7 days following surgery, the rats received microinjections of either quinpirole (0.075–0.3 µg/side) or saline on the day of the experiment, with a total volume of 250 nL per side. In another group of rats, bilateral injections of saline, quinpirole (0.15 µg/side), OXT (3 ng/side), or quinpirole (0.15 µg) + OXT (3 ng/side) were administered in a total volume of 250 nL per side. In a separate experimental setup, rats received bilateral infusions of either saline or raclopride (1 µg/side), a D2R/D3R dopamine receptor antagonist, followed by an infusion of either quinpirole (0.15 µg) + OXT (3 ng/side) or saline vehicle, with a total volume of 250 nL per side. To account for possible volume effects, a subset of rats was infused with 500 nL saline per side in the same experiment. All injections were administered over a 5-min period using an injection cannula (0.20 mm outer diameter, C315I, Plastics One) that extended 1 mm beyond the end of the guide cannula. Two CMA/microdialysis pumps (CMA/Microdialysis, Stockholm, Sweden) were used for the microinjections. After each injection, the cannulas were left in place for 30 s to prevent backflow and to facilitate diffusion into the surrounding tissue. The shock-probe burying test was conducted immediately after the microinjections to assess behavioral responses. Locomotion was measured in an open field to evaluate the general state of the rats after the shock-probe burying test (Pérez de la Mora et al., 2012).

### 2.4 Histology

At the end of the experiments, the animals were euthanized with an overdose of phenobarbital (100 mg/kg i. p.), and their brains were promptly removed from the skull. The brains were fixed in 30% formaldehyde for 24 h and subsequently immersed in sequential sucrose solutions (10%, 20%, and 30%) for at least 24 h each. Coronal slices (50 µm) were obtained using a cryostat (CM 1510-3, Leica Instruments, Nussloch, Germany) and stained with crystal violet to identify the cannula implantation sites within the amygdala.

### 2.5 Shock-probe burying test

The shock-probe burying test was conducted following the protocol described by Treit, 1990 (Fernández-Guasti et al., 2001 and Perez de la Mora et al., 2006). The test involved placing the rats in a plexiglass cage (27 × 16 × 23 cm) with the floor covered by a uniform layer (5 cm) of fine sawdust. An electrified probe (7 cm long, 0.5 cm thick) protruded from one of the cage walls, 2 cm above the bedding, delivering an electric shock (0.4 mA) whenever the rats made contact with it. The electric current was generated by a constant-current shock generator (Lafayette Instruments, Inc.). Once introduced into the cage, the rat’s behavior was recorded for 10 min after the initial behavioral response to the electric shock. Three parameters were measured during the trial: the total time the rat spent burying the probe with its forepaws (burying behavior), the latency of the burying behavior (time elapsed between the first shock and the start of burying), and the number of shocks received by the rat during the test. Burying behavior was considered an indicator of anxiety, burying behavior latency reflected reactivity, and the number of shocks served as an indication of the adverse effects of the electric shocks.

### 2.6 Open-field test

To evaluate the effects of treatments on the general state of the animals, an open-field test was conducted immediately after the shock-probe burying test. Locomotion in this test was carried out in an acrylic box (50/50/30 cm) equipped with photoelectric cells to record the horizontal movements of the animals through the arena (OMNIALVA, Mexico City, Mexico). Each wall contained 10 photoelectric cells separated 5 cm from each other that were located 4.0 cm above the arena. The box is equipped with a software with a PC that quantitatively estimates the number of beam interruptions through the photocells during the locomotion of the animal in the arena and transforms them into arbitrary locomotion events with a sampling frequency of 10 Hz. The illumination level inside the box was 138 lux. At the beginning of the test, the rats were placed in one of the corners of the box and were allowed to explore the arena for 5 min.

### 2.7 *In situ* proximity ligation assay

To investigate changes in the expression and densities of D2R–OXTR heterocomplexes, we conducted the *in situ* PLA following previously described protocols (Borroto-Escuela et al., 2012; Borroto-Escuela et al., 2013a; Borroto-Escuela et al., 2016). We used free-floating formalin-fixed brain sections (30 μm thick) from rats at bregma level (−2.2 mm). The primary antibodies used were goat polyclonal anti-oxytocin receptor (5 μg/mL; ab87312 from Abcam, Sweden) and mouse monoclonal anti-D2R (MABN53, 1:600, Millipore, Sweden). These primary antibodies were previously validated for immunohistochemistry in rat brain tissue and HEK293 cell lines ((Borroto-Escuela et al., 2017b; Borroto-Escuela et al., 2018; Feltmann et al., 2018)). Control experiments for *in situ* PLA were conducted on free-floating formalin-fixed rat brain sections using only one primary antibody (mouse monoclonal anti-D2R, MABN53, 1:600, Millipore, Sweden). The negative control group experiments revealed a 9.8% occurrence of false-positive clusters when compared to the positive control group (100%) in an analysis conducted using images taken from both the central nucleus of the amygdala (CeA) and basolateral amygdala (BLA). To reduce this false-positive signal further, brain sections were incubated in glycine buffer for 45 min at 37°C prior to primary antibody incubation. Similar control experiments were performed in cells transfected with cDNAs encoding only one type of receptor. The *in situ* PLA signal was visualized and quantified using a Leica TCS-SL SP5 confocal microscope (Leica, United States) and the Duolink ImageTool software.

The fixed free-floating rat brain sections, stored at −20°C in Hoffman solution, were washed with PBS and quenched with 10 mM glycine buffer for 20 min at room temperature. Subsequently, the sections were incubated with a permeabilization buffer (10% fetal bovine serum (FBS) and 0.5% Triton X-100, pH 7.4) for 30 min at room temperature. After washing with PBS, the sections were incubated with a blocking buffer (0.2% BSA in PBS) for 30 min at room temperature. Primary antibodies were then applied in suitable concentrations in the blocking solution and incubated with the sections for 1–2 h at 37°C or overnight at 4°C. On the following day, the sections were washed, and the proximity probe mixture (minus and plus probes) was applied and incubated for 1 h at 37°C in a humidity chamber. Unbound proximity probes were removed by washing the slides with blocking solution. The sections were then incubated with a hybridization–ligation solution containing BSA, T4 DNA ligase, Tween-20, NaCl, ATP, and circularization or connector oligonucleotides. After washing with a washing buffer, the sections were incubated with a rolling circle amplification buffer for 100 min at 37°C. Subsequently, the sections were incubated with a detection solution consisting of fluorescent oligonucleotide probes at 37°C for 30 min. Finally, the sections were washed with a washing buffer and mounted onto microscope slides. The DAPI-containing mounting medium was applied, and a coverslip was sealed with nail polish. The sections were stored at −20°C in the dark until confocal microscope analysis.

### 2.8 Image acquisition and analysis

Image acquisition and analysis were performed using a LEICA TCS-SL SP5 confocal microscope and the Duolink ImageTool software. Three different areas of the amygdala were selected, and two randomly chosen magnified sample fields (150 × 150 μm) were used for image acquisition, resulting in a total of six images per animal. Images were inspected to exclude unrepresentable pictures (e.g., those containing blood vessels). Nuclei and PLA signal quantification was performed using the Duolink ImageTool software as previously described (Borroto-Escuela et al., 2013b; Borroto-Escuela et al., 2016). As *in situ* PLA typically provides a resolution in the range of 25–30 nm when secondary IgG antibodies are used (Soderberg et al., 2006; Soderberg et al., 2007), we cannot exclude the possibility that in our current experiments, we may detect D2R–OXTR heteroreceptor complexes and/or the co-location of D2R and OXTR within the same cell membrane nanodomains (hereafter referred to as heterocomplexes). For further details on the strengths and limitations of *in situ* PLA and BRET methods, please see Borroto-Escuela et al. (2013a); Borroto-Escuela et al. (2013b); Gomes et al. (2016).

### 2.9 BRET2 assay

We conducted a BRET2 assay following the previously described procedures (Borroto-Escuela et al., 2011a; Borroto-Escuela et al., 2011b; Romero-Fernandez et al., 2011; Borroto-Escuela et al., 2012). For the BRET2 saturation assay, HEK293T cells were transfected with constant (1 μg) or increasing amounts (0.25–9 μg) of plasmids encoding D2R-Rluc and OXTR-GFP2, respectively. The cells were washed, detached, and resuspended in PBS. Cell suspensions (30 μg protein) were distributed in duplicate into 96-well microplates with a transparent bottom (Corning 3651) for fluorescence measurement or those with a white bottom (Corning 3600) for BRET2 determination. The ligand effects were studied at the BRET50 values obtained from the BRET saturation curve of the vehicle-treated group. In the ligand-induced BRET2 assay, the cells were transfected with plasmids encoding D2R-Rluc (1 μg) and OXTR-GFP2 (0.25 μg) at a ratio determined from the BRET50 values obtained in the vehicle control group during the BRET saturation experiment. Subsequently, the cells were incubated with drugs (D2R and OXTR agonists and antagonists) for 15 min before BRET2 measurement. Coelenterazine-400a (DeepBlue™C substrate, VWR, Sweden) was added, and readings were performed using the POLARstar OPTIMA plate reader (BMG Lab technologies, Offenburg, Germany) with two filter settings (410 nm and 515 nm) for BRET2 determination. The BRET2 ratio was calculated as previously described (Borroto-Escuela et al., 2012). Three independent experiments with eight replicates per group were performed. An increase in the BRET signal after OXT and quinpirole co-stimulation could also be explained by the co-location/co-presence of OXTR and D2R in small endocytic vesicles (Gomes et al., 2016). However, while this possibility cannot be excluded, it is considered less likely in view of the experimental data reported previously (Romero-Fernandez et al., 2013).

### 2.10 Statistical analysis

The results are presented as means ± S.E.M. One-way ANOVA followed by the Dunnett test was used in most cases. However, since the Kolmogorov–Smirnov test indicated that the data from the co-administration of oxytocin and quinpirole did not follow a normal distribution, nonparametric statistics were applied. Medians with their respective interquartile range were used to present the results, and the Kruskal–Wallis statistical procedure followed by Dunn’s test as a *post hoc* test was used for analysis. A significance level of ≤0.05, 0.01, and 0.001 was considered in all cases. Statistical analysis was conducted using GraphPad Prism 6 statistical software. Fluorescent *in situ* PLA signal quantification was performed using the Duolink ImageTool software. *In situ* PLA and BRET2 data were analyzed using GraphPad Prism 5.0.

## 3 Results

### 3.1 Animal experiments

#### 3.1.1 Cannula placements

**FIGURE 1:**
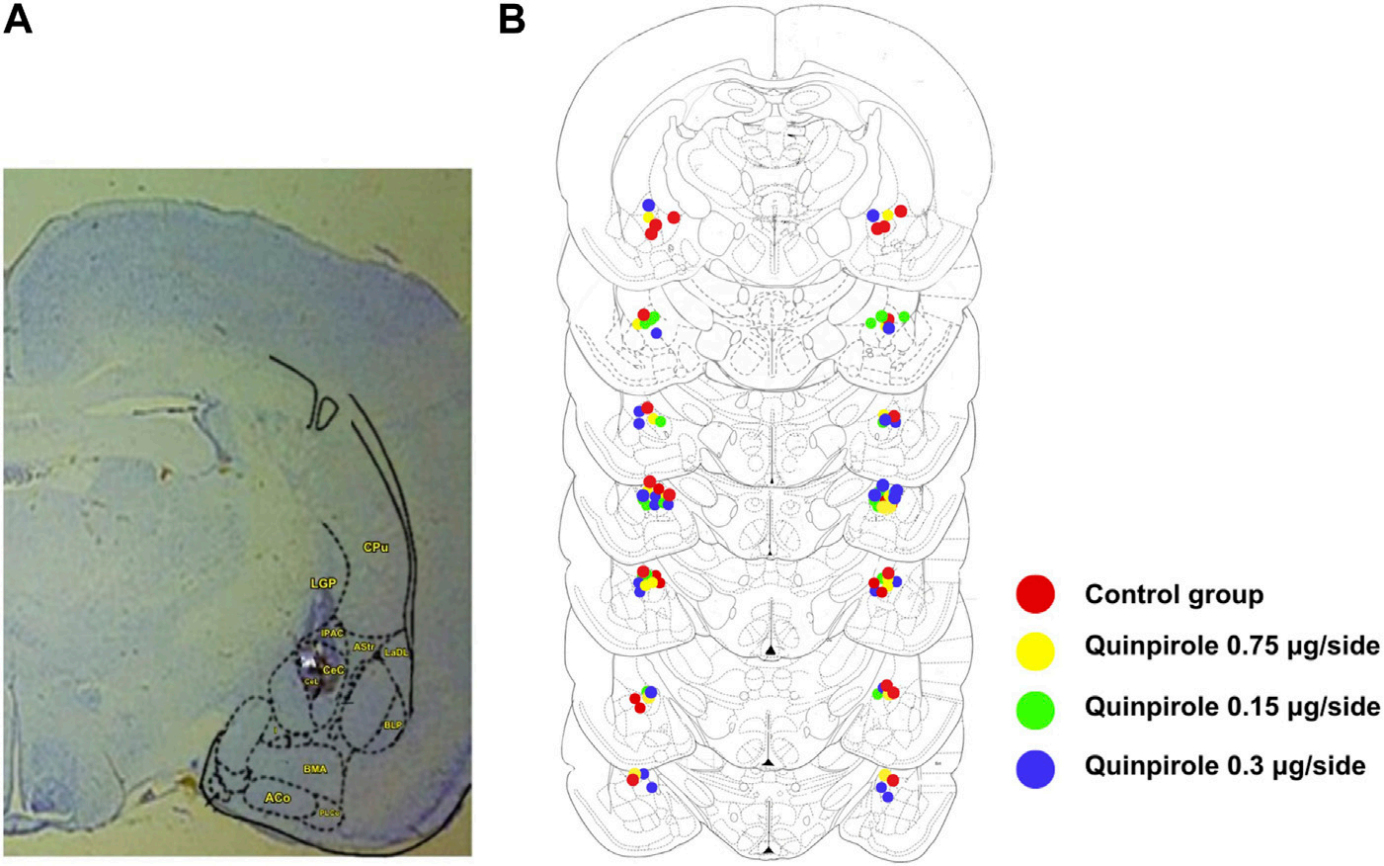
Bilateral cannula placements within the amygdala. **(A)** Panel on the right shows a representative section within the amygdala (Br −2.30) depicting a representative bilateral cannula placement. The star indicates the position of the CeA within the amygdala. **(B)** Schematic representation of the sites of the bilateral cannula placements within the amygdala as verified by histological examination in rats infused with either saline or different doses of quinpirole (0.075–0.3 µg/side). Stereotaxic levels were taken from the rat brain atlas of Paxinos and Watson (1986). Overlapping of cannula placements has been produced in some sections due to the high density of injection sites. A similar distribution pattern of injection sites was observed in all other experiments carried out in this work.

Cannula tips within the amygdala were found for the quinpirole dose–response experiment from AP levels −1.8 to −2.8 mm from the bregma, according to the Paxinos and Watson stereotaxic atlas (1986), with most of them located between −2.12 and −2.56 mm from the bregma. The rats included in the statistical analysis had cannulas either within or very near the CeA, as shown in Figure 1. A similar pattern of cannula tip placements within the brain was found in animals corresponding to all other experiments carried out in this work.

#### 3.1.2 Effects of the bilateral administration of quinpirole into the central amygdala of rats in the shock-probe burying test and their locomotion in the open-field test

**FIGURE 2:**
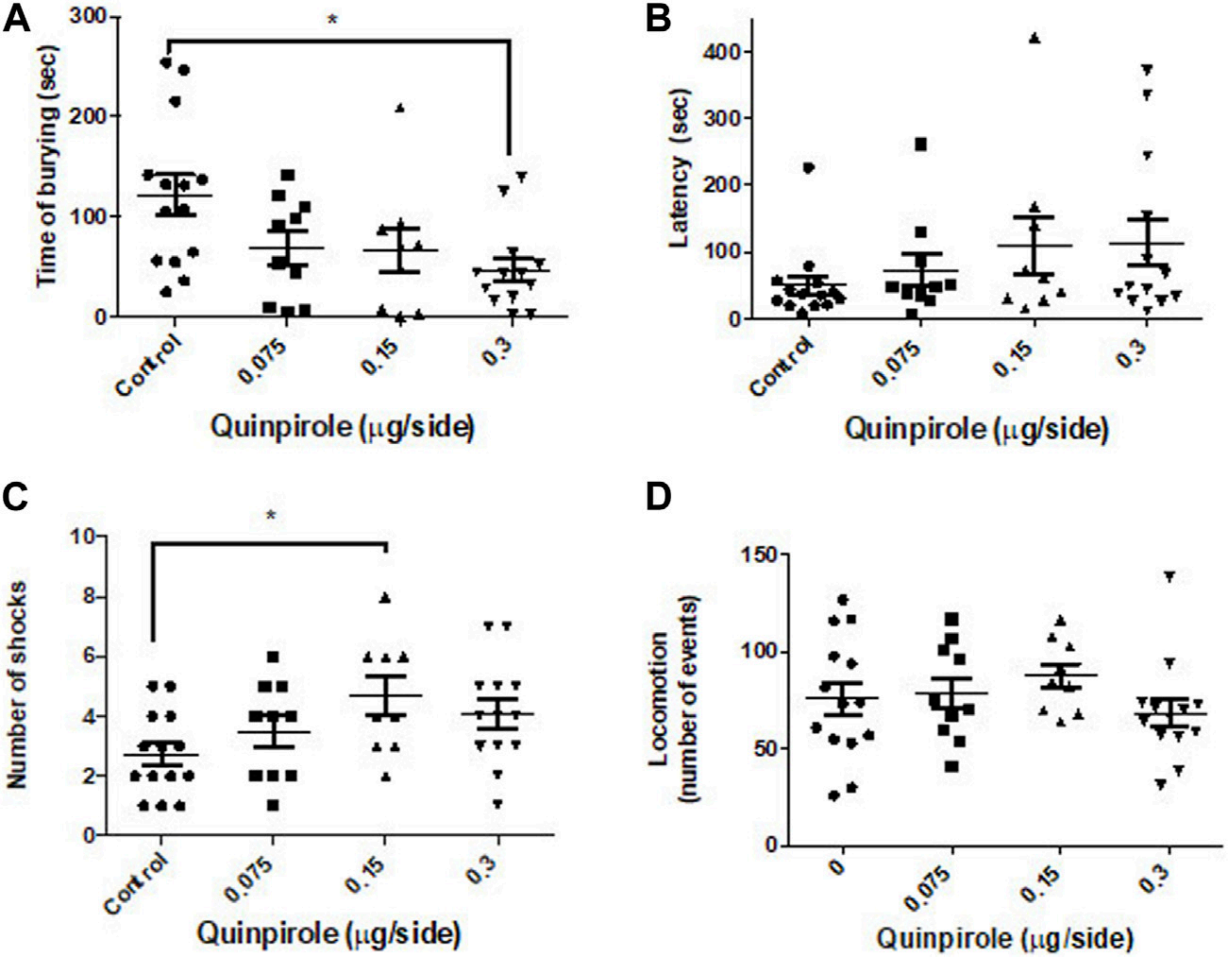
Effects of the bilateral infusion of quinpirole into the central amygdala in the shock-probe burying test and of the locomotion in the open-field test. **(A)** Quinpirole evoked a dose–response decrease in the time that the animals spent burying the electrified probe of the test, whereas **(B)** it does not affect the latency to bury. A modest enhancement **(C)** in the number of shocks received during the test was observed at an intermediate quinpirole dose. **(D)** No effects in the locomotion (events) in the open-field test were noticed. One-way ANOVA was followed by the Dunnett test. *p < 0.05. Control: n = 14; quinpirole 0.075 µg/side: n = 10; quinpirole 0.15 µg/side: n = 9; quinpirole 0.3 µg/side n = 13.

A main effect of quinpirole was observed (one-way ANOVA F_3,42_ = 3.9; *p* < 0.01) on the time rats spent burying the electrified probe of the test. The reductive effect seems to be dose-dependent (Figure 2), although it only reached statistical significance (*p* < 0.01) at the higher dose used concerning the control group (Dunnett’s test). No significant effects were, however, observed in the latency to bury the probe (one-way ANOVA F_3,42_ = 1.2; *p* > 0.05). On the other hand, infusion of quinpirole into the CeA elicited an effect in the number of shocks received during the test (one-way ANOVA F_3,42_ = 2.9; *p* < 0.05), which accounted for a modest enhancement in the number of shocks at the dose of 0.15 μg/side (*p* < 0.05). No effects were, however, found either at a lower (0.075 μg/side) nor at a higher quinpirole dose (0.3 μg/side) relative to the control group (Dunnett’s test). Quinpirole (0.075–0.3 μg/side) failed to affect locomotion in the open-field test (Figure 1) at all the doses used (one-way ANOVA F_3,42_ = 0.92; *p* > 0.5).

#### 3.1.3 Effects of the co-administration of oxytocin and quinpirole into the central amygdala in the shock-probe burying test

**FIGURE 3:**
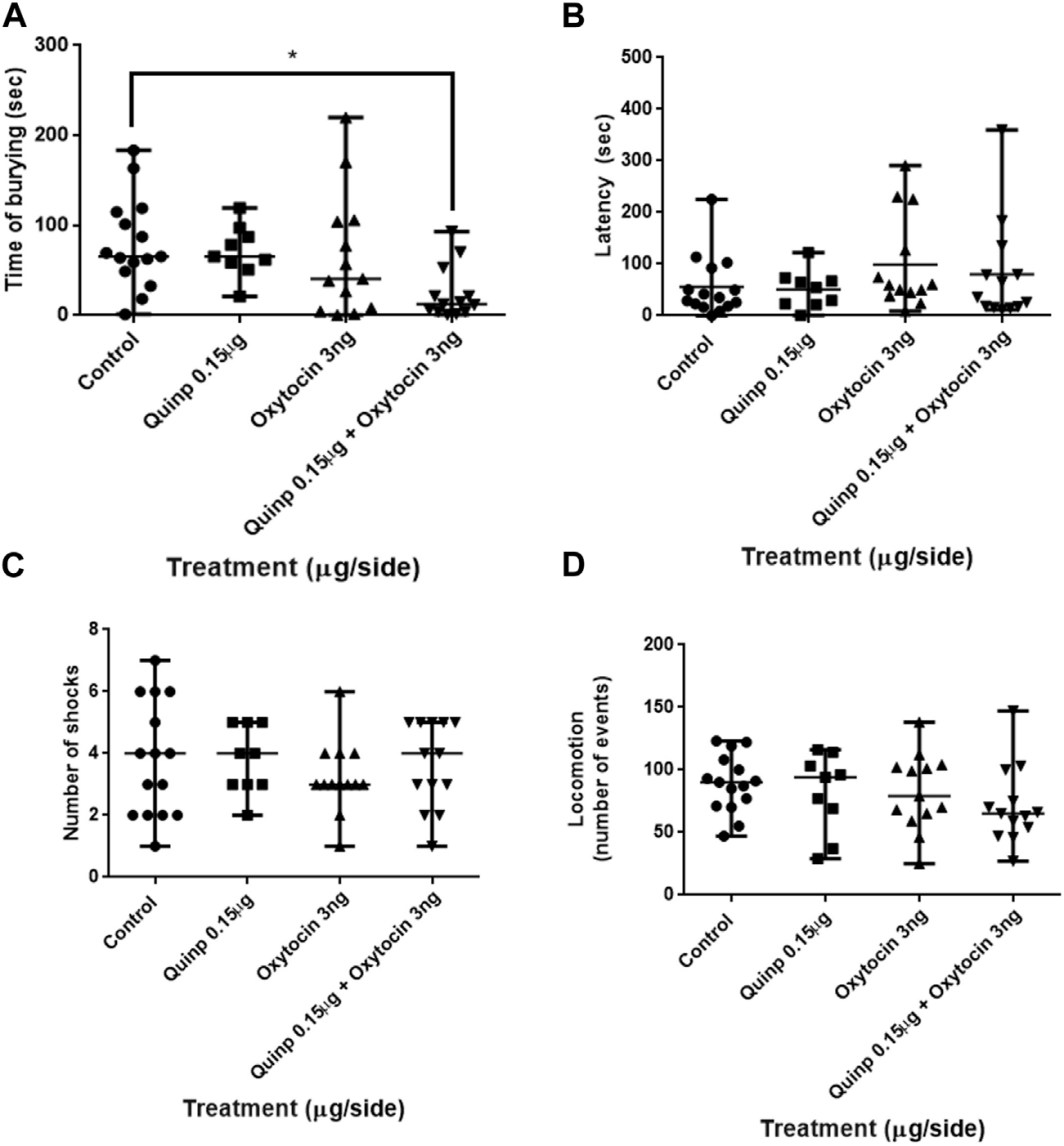
Effects of the co-administration of oxytocin and quinpirole into the central amygdala in the shock-probe burying test and of the locomotion in the open-field test. **(A)** Co-infusion of quinpirole (Quinp) (0.15 μg) + oxytocin (3 ng) evoked a marked decrease in the time that animals spent burying the probe of the test *versus* that of the control group. No effects on **(B)** the latency to the first episode of burying and **(C)** on the number of shocks that the rats received during the test were observed. **(D)** No significant differences in the locomotor activity of any treated group were observed as compared to the control. The Kruskal–Wallis test was followed by the Dunn test. *p < 0.05. Control: n = 15; quinpirole 0.15 µg/side: n = 9; oxytocin 3 ng/side: n = 13; quinpirole 0.15 µg + oxytocin 3 ng/side: n = 13.

Co-administration of sub-optimal doses of oxytocin (3 ng/side) and quinpirole (0.15 μg/side) into the central nucleus of the amygdala resulted in clear-cut effects on the time that the rats spent burying the electrified probe of the test (Kruskal–Wallis test F_3,47_ = 10.92: *p* < 0.05. Dunn’s test analysis showed that although no significant changes in burying behavior were induced by the administration of low doses of both quinpirole and oxytocin, its combination triggered a remarkable decrease in burying activity, relative to the control group (Figure 3). No significant changes were instead observed by either oxytocin or quinpirole alone treatment or its combination on both the latency to the first burying episode (Kruskal–Wallis test F_3,47_ = 2.58; *p* > 0.05) and the number of shocks received by the rats during the test (Kruskal–Wallis test F_3,47_ = 2.58; *p* > 0.05). Likewise, locomotion activity in the open-field test was not significantly affected by any treatment (Kruskal–Wallis test F_3,47_ = 2.58; *p* > 0.05).

#### 3.1.4 Dopamine D2R antagonism on the anxiolytic effects triggered in the shock-probe burying test by the co-administration of oxytocin and quinpirole into the central amygdaloid nucleus

**FIGURE 4:**
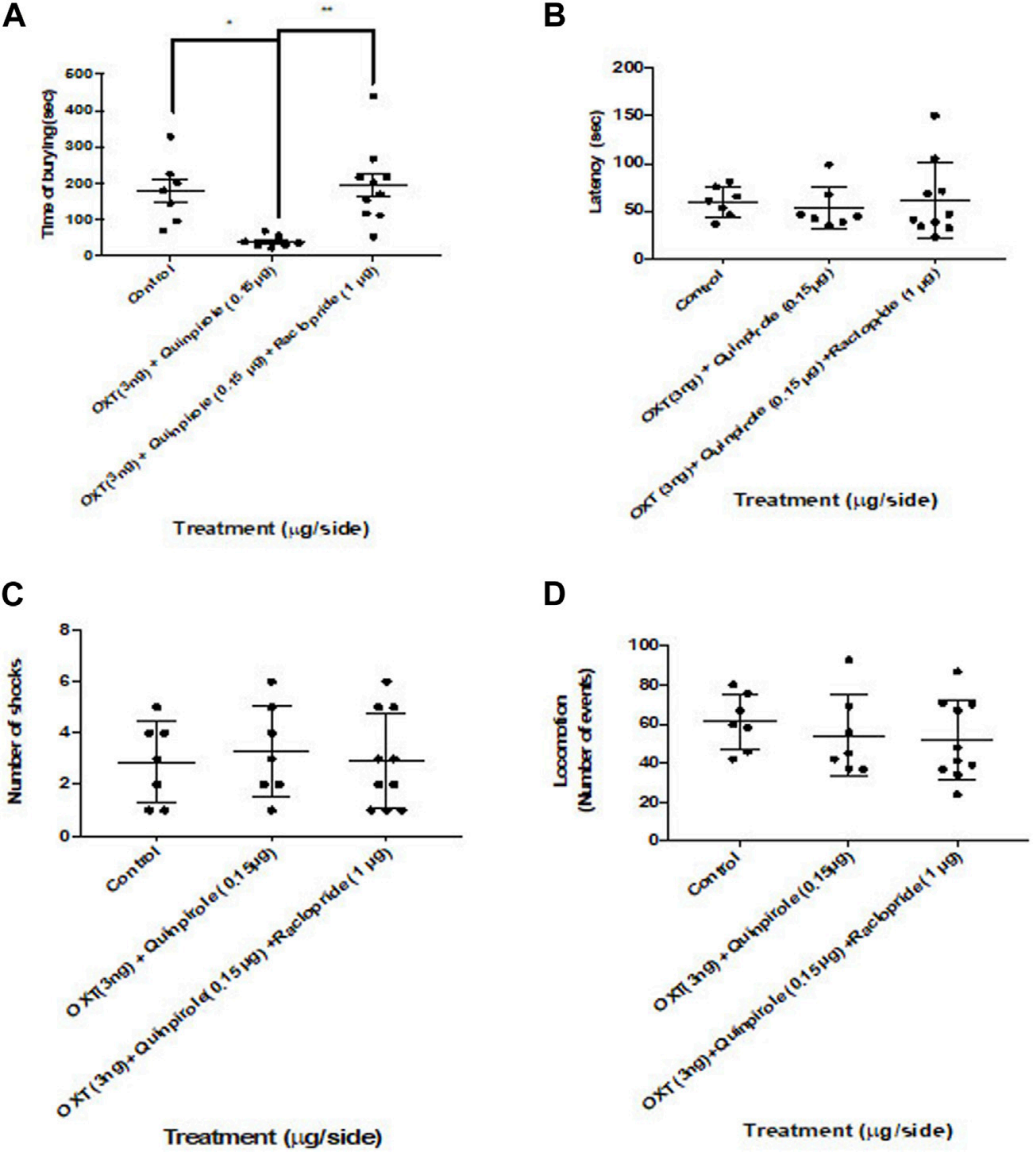
Raclopride blocked the effects of the co-administration of oxytocin and quinpirole into the central amygdala in the shock-probe burying test. **(A)** Raclopride prevented the effects of the co-infusion of quinpirole (0.15 μg) and oxytocin (OXT, 3 ng) in the shock-Probe burying test (*p* < 0.05). **(B)** No changes were, however, observed by the raclopride infusion on the latency to the first episode of burying in this test. No effects were observed in both **(C)** number of shocks and **(D)** locomotion. One-way ANOVA was followed by the Tukey test. *p < 0.05; **p < 0.01. Control: n = 7; oxytocin 3 ng + quinpirole 0.15 µg: n = 7; oxytocin 3 ng + quinpirole 0.15 µg + raclopride 1 µg: n = 10.

To analyze whether dopamine receptor activity was involved in the anxiolytic effects of the co-administration of oxytocin and quinpirole within the central amygdaloid nucleus, we pretreated rats with a previous (15 min) infusion of raclopride within this region (Figure 4). One-way ANOVA (F_2,21_ = 7.7; *p* > 0.05) disclosed statistically significant effects attributable to a dramatic blockade of the co-administration of oxytocin and quinpirole by raclopride on the burying behavior in the shock-probe burying test (Figure 4). No statistically significant changes were noticed in the latency to the first burying episode (Kruskal–Wallis test, F_2,21_ = 0.84: *p* < 0.05) and the number of shocks delivered to the rats during the test. One-way ANOVA was performed (F_2,21_ = 0.87: *p* < 0.05). No effects were also observed in the locomotion in the open-field test. One-way ANOVA was performed (F_2,21_ = 0.59; *p* < 0.05).

### 3.2 Neurochemical experiments

#### 3.2.1 Studies using *in situ* PLA to show the potential existence of D2R–OXTR heteroreceptor complexes in the central amygdala and basolateral amygdala

**FIGURE 5:**
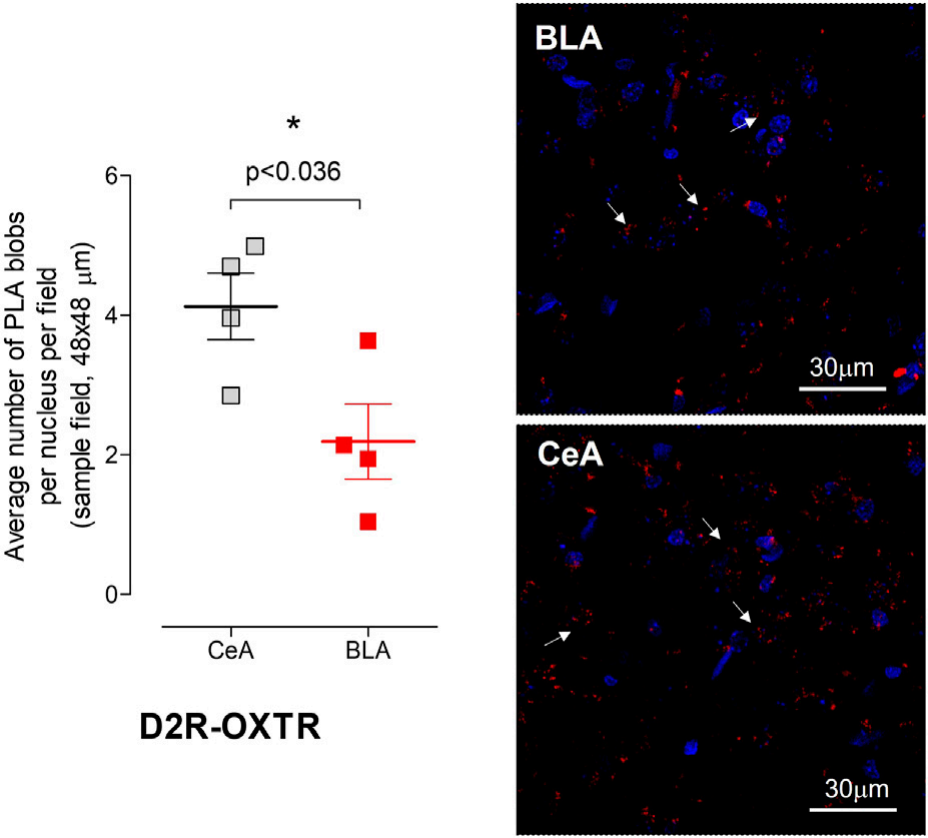
Detection of D2R–OXTR heteroreceptor complexes in the different subfields of rat amygdala by *in situ* proximity ligation assay (*in situ* PLA). The nuclei are shown in blue (DAPI) and the positive PLA puncta/blobs (D2R–OXTR heteroreceptor complexes) in red. (Left panel) Quantitative analysis of the D2R–OXTR heteroreceptor complex expression in the CeA and BLA of the rat brain detected by means of the *in situ* PLA. PLA was quantified as PLA (red puncta/blobs) per nuclei per field by an experimenter blind to the experiment conditions. The analysis (means ± S.E.M.) was performed on images obtained from at least two slices per animal (four rats in total). Statistical analysis was performed by Student’s t-test with Bonferroni correction. The *p*-value of 0.05 and lower was considered significant: *p < 0.05. Bregma 1.00 mm, Scale bar is 30 μm. (Right panels) Representative confocal image of the *in situ* PLA at the level of the CeA and BLA. Some positive PLA clusters/blobs are indicated by an arrow, and the scale bar for this panel is indicated on the right bottom of the panel.

Red PLA puncta were observed in both CeA and BLA, with a significantly higher density in CeA than in BLA (Figure 5). Considering that negative control experiments revealed an approximately 10% occurrence of false-positive clusters when compared to the positive control group (100%) in an analysis conducted using images taken from both the CeA and BLA, and resulting in an average number of PLA puncta per nucleus per sample field of 0.62 ± 0.04, the density of puncta per nucleus per sampled field in the CeA was quantified as moderate (4.12 ± 0.47), whereas in the BLA, it was determined to be low (2.19 ± 0.53). While our *in situ* PLA data indicate the existence of D2R–OXTR heteroreceptor complexes and/or the co-localization of OXTR and D2R receptors within the same cell membrane nanodomains in both the CeA and BLA regions of the amygdala, we conducted additional experiments using proximity-based biophysical techniques (BRET2) in cell lines to gain further insights (shown as follows).

#### 3.2.2 Studies using BRET2 in HEK293 cells on the existence of D2R-OXTR heterocomplexes

**FIGURE 6:**
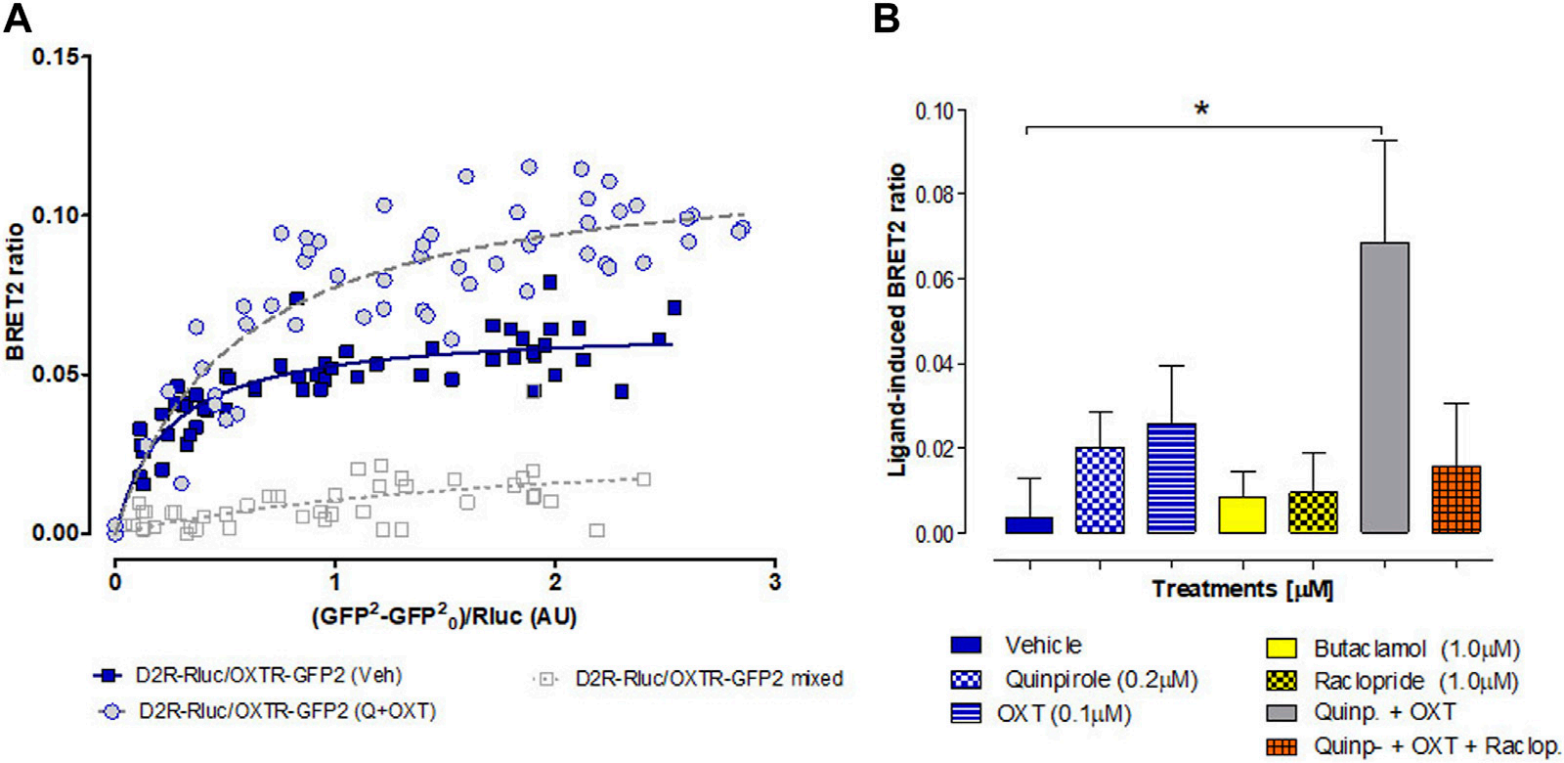
BRET2 analysis was conducted to examine the heterodimerization of D2R and OXTR receptors. **(A)** Transfection of HEK293T cells with a constant DNA concentration of acceptor D2R-Rluc and increasing concentrations of donor OXTR–GFP2 constructs. BRET2 ratio, total fluorescence, and total luminescence were determined as described in the Materials and Methods section. As a negative control, cells individually expressing D2R-Rluc were mixed with cells individually expressing OXTR-GFP2 prior to exposure to coelenterazine-400a. The *X*-axis represents the fluorescence value obtained from GFP2, normalized with the luminescence value of D2R-Rluc expression 10 min after coelenterazine incubation. Ten saturation curves were plotted, and a nonlinear regression equation assuming a single binding site was used to fit the curves. The D2R–OXTR curve exhibited a better fit to a saturation curve compared to a linear regression, as demonstrated by the F-test (*p* < 0.01). This finding contrasts with the mixed pool of cells expressing D2R-Rluc and OXTR-GFP2. The data presented are the mean ± S.E.M., with n = 15–20, performed in triplicate. **(B)** Evaluation of the effects of 20 min of stimulation with different D2R and OXTR agonists and antagonists, as well as combined treatments, on the BRET ratios for D2R–OXTR heteroreceptor complexes. The ratios are expressed as the mean ± S.E.M. from at least six experiments. Combination treatment with quinpirole and oxytocin shows statistically significant difference compared with the vehicle group, one-way ANOVA, Bonferroni post-test.

To explore the potential direct interaction between D2Rs and OXTRs and investigate ligand-induced changes in complex reorganization, we conducted BRET2 assays on HEK293T cells. These cells were co-transfected with a constant amount of the D2R-Rluc construct, while increasing the concentrations of the OXTR-GFP2 plasmid. The objective of this study was also to validate previous experiments conducted on rat brain tissue using *in situ* PLA (Figure 5). As shown in Figure 6A, we observed significant, strong, and saturable BRET2 signals for the D2R–OXTR complex under vehicle-treated conditions (BRET2max of 0.065 ± 0.002). Notably, the BRET2 signal reached even higher maximal values (BRET2max of 0.119 ± 0.007) following a combination treatment with oxytocin (100 nM) and quinpirole (200 nM). The BRET2 signaling in co-transfected cells exhibited a hyperbolic response curve with increasing concentrations of the OXTR–GFP2 fusion construct, reaching an asymptote at the highest concentrations tested. Conversely, when we examined a mixture of singly expressing D2R-Rluc cells and OXTR-GFP2 cells (Figure 6A, mixed cells), a quasi-linear curve was observed, resulting in a nonspecific BRET2 signal.

The observed changes in the BRET signal suggest alterations in dimer formation (as indicated by the significant increase in BRETmax values) or conformational changes within pre-existing complexes. Therefore, we further investigated the effects of ligands on D2R–OXTR heteroreceptor complexes. The cells co-expressing a constant amount of D2R-Rluc and OXTR-GFP2 were incubated with various D2R and OXTR agonists and antagonists for 20 min. Surprisingly, none of the administered ligands consistently induced changes in the BRET2 ratio, suggesting that the dimers are formed constitutively and that the activation or inhibition of each protomer by agonist or antagonist treatments does not affect their oligomerization state (Figure 6B). However, a significant increase in the BRET ratio signal compared to the vehicle control group was observed when a combination treatment with D2R and OXTR agonists was applied (*p* < 0.05).

## 4 Discussion

In the current work, we have further studied the feasibility that DA–OXT receptor interactions having relevance for anxiety may exist in the amygdala and involve the formation of D2R–OXTR heterocomplexes having bilateral stimulatory receptor–receptor interactions. Our findings strongly indicate the existence of D2R–OXTR heteroreceptor complexes and/or populations of OXTRs and D2Rs co-located within the same cell membrane nanodomains. In this region, they may be characterized by bidirectional allosteric receptor–receptor interactions (Romero-Fernandez et al., 2013; de la Mora et al., 2016).

Thus, in line with our previous results (de la Mora et al., 2016), infusion of quinpirole, a D2R-like agonist, into the amygdala (0.075–0.3 µg/per side) triggered anxiolytic effects in the shock-probe burying test in a dose-dependent way. Such effects are suggested from the infusion of the lowest dose of quinpirole and reached clear statistical significance when the highest dose of this compound was administered. Furthermore, the simultaneous infusion of noneffective doses of quinpirole (current work) and OXT (de la Mora et al., 2016) also elicited anxiolytic effects. These effects were most importantly prevented in the current work by the prior intra-amygdaloid infusion of raclopride, a selective D2R/D3R antagonist.

It is worth mentioning that the aforementioned results were obtained using the shock-probe burying test, a well-validated animal model of anxiety (Pinel and Treit, 1978; Treit, 1990; Everts and Koolhaas, 1999; Perez de la Mora et al., 2006; McNeal et al., 2018). The reduction in burying behavior (the most sensitive measure of anxiety observed in our work) is consistent with the triggering of anxiolytic effects. No changes in latency to burying were observed in any of our experiments, and only a modest but otherwise not reproducible increase was observed in the number of shocks elicited by a single intermediate dose of quinpirole (compare Figures 2 vs. Figure 3; Figure 6) in this work. On the other hand, our results showing that no effects on locomotion were elicited in any of our experiments in which quinpirole was administered suggest that no locomotor effects of this drug were involved in its anxiolytic effects.

The results of our current experiments fully agree with the important role that the amygdaloid dopaminergic neurotransmission has in the modulation of both conditioned fear (see for reviews (Pezze and Feldon, 2004)) and fear and anxiety (de la Mora et al., 2010; Zarrindast and Khakpai, 2015). However, in the work of Bananej et al. (2012), the intra-BLA D2R stimulation by quinpirole (Eilam and Szechtman, 1989) elicited anxiogenic effects in the elevated plus maze test, and anxiolytic effects were described here in the shock-probe burying test following its infusion within the CeA. Furthermore, although both anxiogenic and lack of effects have been reported in our work (Perez de la Mora et al., 2012) following the D2R blockade within the CeA in the shock-probe burying test and the elevated plus maze test, respectively, both anxiolytic and anxiogenic effects have been described by t Zarrindast et al. (2011) when the amygdaloid DA receptors were blocked within the BLA by sulpiride, also a well-known D2R antagonist (Seeman and Van Tol, 1994). The reason for these experimental differences is quite unclear, but it may involve as a part of the genetic background of the experimental animals used in each laboratory, differences in both the cannulation sites (region administered), the extent of the drug diffusion, and the anatomical targets finally reached. Furthermore, differences in the neural pathways involved in each behavioral test and their relative contribution to the effects of the drugs used on either auto- or post-synaptic D2Rs may have also played a decisive role in explaining these differences.

Oxytocin, on its side, has been widely demonstrated to be a critical modulator of fear and anxiety (Jurek and Neumann, 2018), which in the vast majority of cases, elicits anxiolytic effects (see Jang et al. (2021); Chun et al. (2022)). Thus, in agreement with this, results from our laboratory (de la Mora et al., 2016) showed that oxytocin infusion (above 25 ng/side) into the CeA triggered anxiolytic effects in rats when using the shock-probe burying test. Such effects were abolished by the simultaneous administration of OTA, an OXTR antagonist (Elands et al., 1988). However, in contrast to the findings of Laszlo et al. (2016); Laszlo et al. (2020), no effects of oxytocin on anxiety were observed in the elevated plus maze test in our studies (unpublished data) and those of Bale et al. (2001). Although the reason for such divergent effects is unclear, but it may be linked to a different maze design (Hogg, 1996) employed in each of those experiments. It is, however, of considerable interest that both the co-injection of oxytocin with raclopride (de la Mora et al., 2016) and the previous raclopride administration (Laszlo et al., 2020) prevented the oxytocin-induced anxiolytic effects, suggesting a possible interaction between the OXTR- and the D2R-mediated neurotransmission on the modulation of anxiety. Such a suggestion has been further supported by the triggering of anxiolytic effects elicited in this work by simultaneously injecting ineffective doses of both oxytocin and quinpirole and its prevention by the previous amygdaloid D2R blockade with raclopride. Furthermore, such interaction seems to be of a facilitatory nature since, as indicated before, ineffective doses of both quinpirole and oxytocin potentiate each other to elicit anxiolytic effects.

The way OXTR- and D2R-mediated neurotransmissions interact to elicit facilitatory interactions with relevance for anxiety within the amygdala is not entirely clear. However, in the current work, D2R–OXTR heterocomplexes were indicated to exist using *in situ* PLA, in moderate densities in the central amygdala, and in low densities in the basolateral amygdala. Previously, heterocomplexes formed by D2R and OXTR protomers were shown to take place in the nucleus accumbens shell and the dorsal striatum of the rat in terms of D2R signaling and recognition (Romero-Fernandez et al., 2013; Borroto-Escuela et al., 2022). In the current study, enhanced allosteric D2R–OXTR interactions based on the behavioral findings were indicated to exist in the central amygdala within GABAergic interneurons and GABAergic projection neurons. These neurons are preponderant in this nucleus (Sun and Cassell, 1993), contributing to anxiolytic effects induced via the D2R and OXTR protomers.

We propose that in the basolateral amygdala (Perez de la Mora et al., 2012) as well as in the central nucleus of the amygdala, including the contralateral (CeL) and centro medial (CeM) amygdala, D2R–OXTR heterocomplexes may exist. These potential D2R–OXTR heterocomplexes may predominantly modulate the signaling of GABA interneurons and projection neurons. According to these assumptions, the activity of amygdaloid GABA projection neurons toward the brainstem can reach the brainstem, including the hypothalamus, periaqueductal gray, and locus coeruleus. They could significantly depend on the integration with activity in the glutamate excitatory drive, inhibitory GABA interneurons, and the inhibitory activity of the D2R protomer within the potential D2R–OXTR heterocomplex present in these GABA projection neurons. Additionally, it should be considered that GABA interneurons and GABA projection neurons in the target regions can mutually modulate each other’s effects. Therefore, it is proposed that an enhanced activity in the inhibitory amygdala GABA projection neurons to the brainstem could be an important mechanism involved in inhibiting local GABA interneurons in this region, setting free increased activation in, e.g., monoamine ascending pathways from the lower brainstem and the hypothalamus. These ascending brainstem neurons like the noradrenergic and cholinergic neurons may markedly alter the modulation of limbic system signaling, including the amygdala in a way to reduce stress and anxiety. For future work, it is also essential to understand the impact of pre- and post-synaptic actions on these brainstem projections on the amygdaloid neurons and their heterocomplexes, like the potential D2R–OXTR heterocomplexes.

Furthermore, it may also be possible that in agreement with de la Mora et al. (2016), the potential receptor–receptor interactions may involve an enhancing effect of D2R–OXTR heterocomplexes on CREB, MAPK, and PLC signaling pathways since in co-transfected HEK293 cells, allosteric reciprocal D2R–OXTR interactions have been proven to exist and signal through these pathways. In our current study, we successfully demonstrated the presence of D2R–OXTR heteromers in HEK293 cells using the BRET2 method. Specifically, through this approach, we were able to observe and measure enhanced BRET2max signals following the combined agonist stimulation of D2R and OXTR protomers. These findings provide robust indications for the existence of D2R–OXTR heterocomplexes and highlight their potential functional significance in cellular signaling. An increase in the BRET signal after OXT and quinpirole co-stimulation could also be explained by the co-location/co-presence of OXTR and D2R in small endocytic vesicles (Gomes et al., 2016). However, while this possibility cannot be excluded, it is considered less likely in view of the experimental data reported previously (Romero-Fernandez et al., 2013).

## 5 Concluding remarks

Our behavioral results obtained with the use of the shock-probe burying test, an unconditioned paradigm of anxiety, unveiled the potential presence of bilateral anxiolytic D2R–OXTR receptor interactions within the amygdala having reciprocal enhanced anxiolytic effects on anxiety. Such interactions may occur through allosteric changes occurring at the interface of the potential D2R–OXTR heterocomplexes. Given the paramount role of the amygdala in the modulation of anxiety, considerable efforts should be devoted either to favor the formation of these heterocomplexes and/or to stabilize them when designing novel anxiety treatment approaches.

## Data Availability

The original contributions presented in the study are included in the article/supplementary materials, further inquiries can be directed to the corresponding authors.
